# Adaptive Avoidance of Reef Noise

**DOI:** 10.1371/journal.pone.0016625

**Published:** 2011-02-04

**Authors:** Stephen D. Simpson, Andrew N. Radford, Edward J. Tickle, Mark G. Meekan, Andrew G. Jeffs

**Affiliations:** 1 School of Biological Sciences, University of Bristol, Bristol, United Kingdom; 2 Australian Institute of Marine Science, The UWA Oceans Institute, Crawley, Australia; 3 Department of Marine Science, University of Auckland, Auckland, New Zealand; University of California Davis, United States of America

## Abstract

Auditory information is widely used throughout the animal kingdom in both terrestrial and aquatic environments. Some marine species are dependent on reefs for adult survival and reproduction, and are known to use reef noise to guide orientation towards suitable habitat. Many others that forage in food-rich inshore waters would, however, benefit from avoiding the high density of predators resident on reefs, but nothing is known about whether acoustic cues are used in this context. By analysing a sample of nearly 700,000 crustaceans, caught during experimental playbacks in light traps in the Great Barrier Reef lagoon, we demonstrate an auditory capability in a broad suite of previously neglected taxa, and provide the first evidence in any marine organisms that reef noise can act as a deterrent. In contrast to the larvae of species that require reef habitat for future success, which showed an attraction to broadcasted reef noise, taxa with a pelagic or nocturnally emergent lifestyle actively avoided it. Our results suggest that a far greater range of invertebrate taxa than previously thought can respond to acoustic cues, emphasising yet further the potential negative impact of globally increasing levels of underwater anthropogenic noise.

## Introduction

Across the animal kingdom, acoustic information is frequently used in orientation, habitat selection and predator avoidance. Marine coastal habitats, for example, are characterised by a high level of biological and abiotic noise, and coral reefs are particularly noisy due to high densities of resident shrimps, urchins and fishes [1], [2]. Underwater, sound has two major components: in the acoustic nearfield (confined to an area within 1 or 2 wavelengths) particle velocity dominates, while in the acoustic farfield, the propagating pressure wave component dominates [3], [4]. These acoustic components are detected by animals in two ways: sensory hair-like receptors are used to detect one-way particle displacement of water in the nearfield, whereas membranous receptors are used for the detection of farfield two-way particle oscillations. While these sensory mechanisms are well understood for fish and marine mammals, there is a relative paucity of information on whether aquatic invertebrates can also detect and utilise acoustic cues.

Many benthic marine organisms undergo an early developmental stage at sea and must settle to suitable habitat for juvenile and adult life [5]. A number of studies have shown that settlement-stage larvae of a broad range of coral reef fishes can detect, and are attracted to, the noises of coral reefs [6]–[9]. There is also evidence that the larvae of some crabs and fishes in temperate waters use acoustic cues from urchin-dominated reefs to detect and locate settlement sites (see [4]). In addition to species that settle to reefs, the surrounding waters are home to a diverse community of free-swimming organisms (many of them crustaceans) that do not dwell in reef habitats; rather, their chances of survival are likely to be greatly enhanced by avoiding such areas of high potential predation risk [10], [11]. Selection might therefore be expected to act on these species to evolve an ability to detect and avoid reef noise, but this possibility has never been explored.

Here we use experimental playbacks and light traps in the waters of the Great Barrier Reef lagoon to test the responses to coral reef noise of a broad suite of tropical crustaceans with a range of life-history strategies. We predict that larval stages of taxa that inhabit reefs as adults will, if they can detect the sound, be attracted to reef noise. In contrast, we predict that both pelagic taxa (those that remain in the water column throughout their lives) and nocturnally emergent taxa (those that ascend into the water column at night, but spend the day hidden in soft benthic sediment) will, if they are capable of detecting it, be deterred by reef noise.

## Methods

### Ethics Statement

All work was carried out under the guidelines of the Ethics Committees of the Australian Institute of Marine Science and Lizard Island Research Station, and with permission from the Great Barrier Reef Marine Park Authority, Australia.

**Figure 1:**
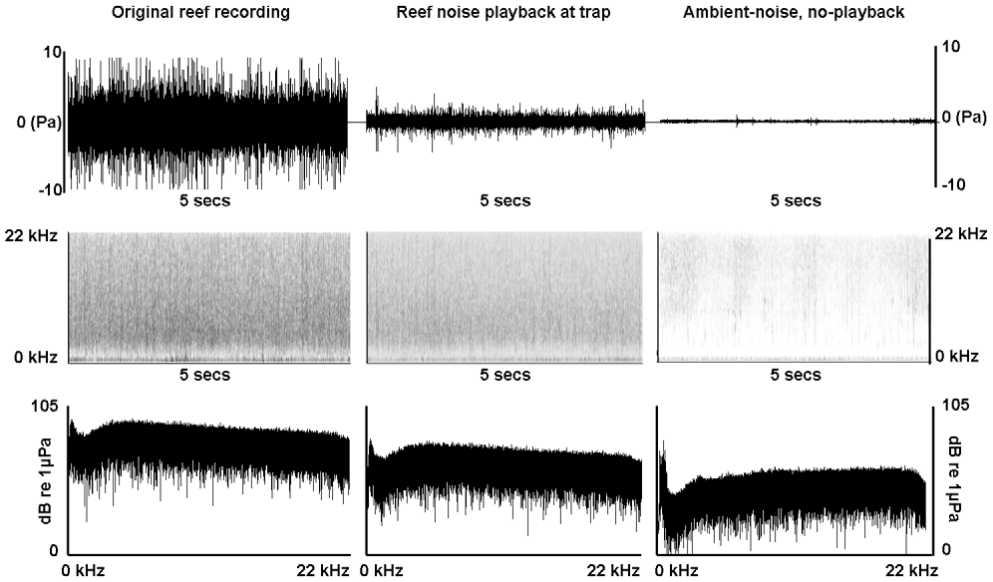
Acoustic representation of the reef recording and experimental conditions. Reef recording used (left) and acoustic conditions at the trap with (centre) and without (right) playback. Top row: time signal; Middle row: spectrogram; Bottom row: spectral levels.

The study was conducted between November 2001 and January 2002 at Lizard Island Research Station (14°40′S 145°28′E), Great Barrier Reef, Australia. We sampled for 34 nights using a pair of light traps which consisted of an 8 W fluorescent light housed in a clear Perspex box with one 1×25 cm entry slit on each side [12]. These traps are highly effective for sampling mobile, photopositive fishes and crustaceans [13]. Traps were attached to permanent moorings, 180 m apart and >500 m from shore, in 10–15 m depth of water over sand at one of three locations (two in front of the Research Station and one at Coconut Beach, location determined by prevailing weather conditions; [7]). Each night, one light trap was randomly allocated a sound system while the other had a dummy rig attached to eliminate modification of the catch arising from additional floating objects. Our sound system consisted of a waterproof barrel containing a 12 V marine battery, 70 W amplifier and portable CD player, playing back reef noise through an underwater speaker (UW-30, frequency response 0.1 to 10 kHz, University Sound, Buchanan) fixed 1 m below the water surface and 1 m from the light trap. We used a 4-min recording of reef noise (Fig. 1), made using a calibrated Clevite CH17 hydrophone (flat response between 1.1–15 kHz, 5 dB drop-off below 1 kHz), a RANRL preamplifier (40 dB gain) and a Sony TCD-D7 digital tape deck. The recording was from a mid-shelf reef on the Great Barrier Reef which is similar to the reefs surrounding Lizard Island, and captured the dusk chorus of biological noise recorded during the new moon phase, consisting of a chorus of pops made by nocturnal fishes together with a higher frequency (2.5– >20 kHz) but lower intensity background crackle produced by snapping shrimps as well as other feeding, movement and calling sounds. The recording was played back throughout the night on a continuous loop at a broadband (root mean square, rms) playback level set at 104 dB re 1 µPa, which ensured that the trap without playback did not receive additional noise above local ambient sound levels (measured at dusk at rms level of 93.8 dB re 1 µPa; Fig. 1). Using p =  ρ*c*v (where p =  pressure in Pa, ρ =  water density in kg m^−3^, *c* =  speed of sound in m s^−1^, and v =  particle velocity in m s^−1^ [3]), the particle velocity near to the speaker during playback would be 6.68×10^−8^ m s^−1^.

Traps were deployed at dusk and retrieved at dawn. The catch was preserved in 70% ethanol and the crustaceans separated from the fish prior to categorisation and counting. To ensure that the numbers of captured crustaceans had not been modified by fish predation in the traps, we dissected 90 pelagic baitfish and 90 settlement-stage reef fish selected evenly from the two sound treatments and randomly from nine different nights. We found no fish with freshly consumed crustaceans in their mouth, throat or stomach, so rule out the possibility that differential predation drives any differences in crustacean catches.

The vast majority (99.3%) of the nearly 700,000 crustaceans caught were divided into 15 reliably distinguishable categories using a dissecting microscope; the remainder were not included in analyses. Any categories for which more than 50% of nights produced no catch in both traps (implying that there were no individuals of this category in the location on that occasion) were discarded prior to analysis. This criterion eliminated Euphausiacea, Palinura and Stenopodidae. Remaining categories for which the mean nightly catch was less than 200 individuals were also discarded. This criterion eliminated Isopoda, Sergestidae and Stomatopoda. We therefore had nine categories for statistical analysis: two larval developmental stages of reef-settling Brachyura (zoea and megalops), two pelagic taxa (Copepoda and Hyperiidea), and five taxa that tend to be mostly nocturnally emergent (Caridea, Cumacea, Gammaridea, Mysidae and Ostracoda).

Data were analysed using generalised linear mixed models (GLMMs) to allow the inclusion of random as well as fixed terms and thus control for repeated measures from the same trap locations and paired trapping on the same night. For each crustacean category, we used a separate GLMM with a Poisson error distribution and a log link function to examine how sound treatment (reef noise playback; ambient-noise, no-playback control) affected number of individuals caught in the trap. Each GLMM was based on 68 catch totals from paired trapping on 34 nights at three different locations. Variance components were estimated using the Restricted Maximum Likelihood (REML) method, and random terms were retained unless the variance component was found to be zero (and hence their removal did not influence the analysis). The significance of fixed terms was determined using the Wald statistic, which approximates the χ^2^ distribution. In each model, we included trap pair (i.e. the two traps from the same night) nested in trap location as a random term. Statistical analyses were two-tailed and were conducted in Genstat (13th edition, Lawes Agricultural Trust, Rothampstead, Harpenden, UK).

## Results

Of the approximately 691,000 individuals analysed statistically, 9.3% were developmental stage reef-settling Brachyura (megalops: 5.7%; zoea: 3.6%), 18.9% were pelagic taxa (Copepoda: 1.7%, Hyperiidea: 17.2%), and 71.8% were taxa that tend to be mostly nocturnally emergent (Caridea: 2.4%; Cumacea: 12.6%; Gammaridea: 9.5%; Mysidae: 42.1%; Ostracoda: 5.3%).

**Figure 2:**
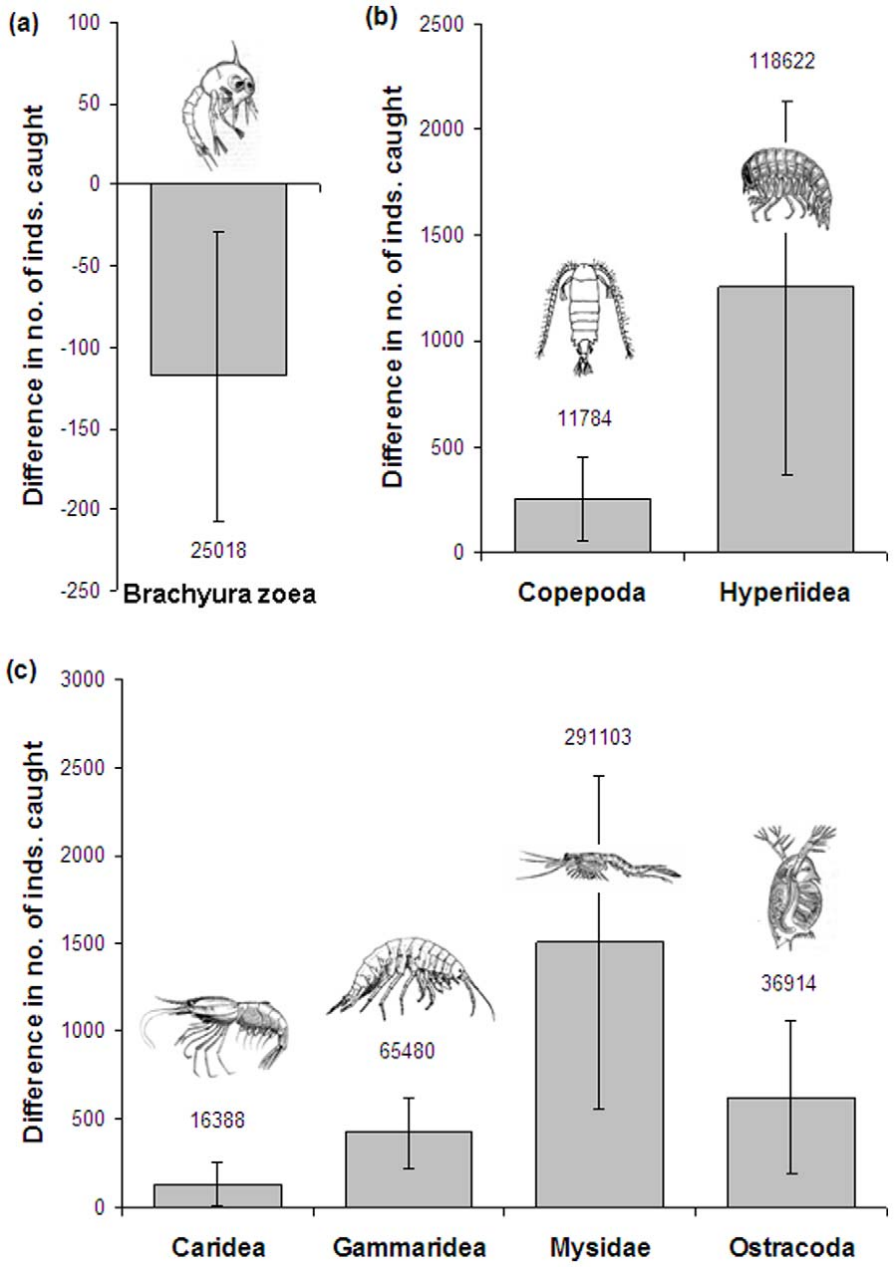
Catches of crustacean taxa in light traps with and without reef noise playback. Mean ± se difference in total number of individuals of (a) larval developmental stage of reef-settling Brachyura, (b) pelagic taxa and (c) nocturnally emergent taxa; negative values indicate greater numbers in noise traps, positive values indicate greater numbers in control traps. N = 34 pairs of traps on separate nights. Numbers above or below bars indicate total number of individuals sampled.

There was no significant difference in the number of brachyuran megalops caught depending on sound treatment (GLMM: Wald statistic = 3.05, df = 1, P = 0.085), but brachyuran zoea were caught in significantly higher numbers in traps playing back reef noise compared to control traps (Wald statistic = 5.63, df = 1, P = 0.021; Fig. 2a).

Both pelagic taxa were found in significantly greater numbers in the control traps compared to those with reef noise playback (Copepoda: Wald statistic = 17.22, df = 1, P<0.001; Hyperiidae: Wald statistic = 22.31, df = 1, P<0.001; Fig. 2b). Of the five taxa that tend to be mostly nocturnally emergent, Cumacea did not show a significant response to reef noise playback (Wald statistic = 2.79, df = 1, P = 0.100), but Caridea (Wald statistic = 18.89, df = 1, P<0.001), Gammaridea (Wald statistic = 24.39, df = 1, P<0.001), Mysidae (Wald statistic = 16.88, df = 1, P<0.001) and Ostracoda (Wald statistic = 52.87, df = 1, P<0.001) were all significantly more common in control traps compared to those playing back reef noise (Fig. 2c).

## Discussion

Our study demonstrates that a wide range of crustaceans with a variety of habits and life-history strategies are capable of detecting and responding to acoustic information. Previous evidence for such behaviour is restricted to the larval stages of a subset of taxa (mostly crabs) that recruit to reef habitat [14], [15]. Furthermore, we provide the first experimental evidence in any marine organisms that taxa found in the proximity of reefs, but which do not settle to them, actively avoid reef noise. These taxa can potentially benefit from such avoidance behaviour because reefs are home to a wide variety of mobile and site-attached predators that feed there both day and night [10], [11]. Exploitation of the rich resources available in inshore waters must be balanced against this risk of predation, and mechanisms for optimising this trade-off should be selected over evolutionary time. Sound provides an excellent indicator of the direction and proximity of reefs [16] and there is a clear survival benefit in utilising acoustic information to detect and avoid such hazardous locations.

As predicted, zoea, the pre-settlement larval stage of Brachyura, were attracted to reef noise. This is consistent with findings from temperate waters (e.g. [10]), but provides the first evidence for such a response in the tropics. The ability of zoea to use acoustic cues to help locate and remain within the proximity of suitable settlement habitat could be critical for recruitment success, despite any increased predation pressures. In contrast to zoea, megalops, the larval settlement stage of Brachyura, appeared not to be attracted to reef noise. One possible explanation for this is that any attraction of these late-stage larvae to reef noise (see [4]) was countered by a downward-swimming settlement response induced by the same noise (see [17]), causing some megalops to move away from those traps coupled with noise playback.

In addition to a diverse suite of biological noises, the soundscape in shallow water environments is influenced by local bathymetry, seabed characteristics and surface conditions [3]. These factors combine to determine the distance over which reef noise propagates above ambient offshore levels [16]. Since hearing in crustaceans is poorly understood, and may be in the farfield via specialised acoustic pressure detectors [18] or limited to the nearfield through particle motion detection, a broad taxonomic investigation of hearing mechanisms and thresholds is needed to enable predictions of the likely distance of detection of reef habitats by crustaceans.

Coral reef noise is heterogeneous in time and space, and these differences relate directly to habitat type [19], [20] and the density of fishes [21]. The reef sounds we played back were largely comprised of a background crackle generated by snapping shrimp and the pops, grunts and gurgles of nocturnal fishes (predominantly Holocentriae and Apogonidae). More work, potentially using *in situ* choice chambers [22], [23], is needed to determine the level of selectivity of crustaceans to different sounds, and whether specific sounds (e.g., predatory fish vocalisations) or general broadband noise levels drive their directional behaviour.

There is much recent concern that natural marine soundscapes are being modified or dominated in some places by anthropogenic noise arising from, for example, shipping and small boats, drilling and mining, seismic surveys and offshore construction [24]. In modified acoustic environments, this can lead to masking of naturally important cues [25] which, given our results, may mean that reef-settling crustaceans detect suitable adult habitat over smaller distances, and non-settling crustaceans are less able to detect and avoid potentially dangerous reef environments. In addition, a recent study has demonstrated that, following several hours of exposure, reef fish larvae can become attracted to artificial sounds that would normally be avoided [22]. If this was also the case for crustaceans, anthropogenic noise could lead to maladaptive behaviour by invertebrate taxa that underpin critical foodwebs and fisheries. Our study, demonstrating detection and ecologically relevant use of reef noise in a broad suite of tropical crustaceans, suggests that the use of sound for orientation is far more widespread than previously thought, and highlights the need for further research into the impact of anthropogenic noise throughout marine ecosystems.
